# Prevalence and its associated psychological variables of symptoms of depression and anxiety among ovarian cancer patients in China: a cross-sectional study

**DOI:** 10.1186/s12955-017-0738-1

**Published:** 2017-08-17

**Authors:** Chun Li Liu, Li Liu, Yi Zhang, Xiao Ze Dai, Hui Wu

**Affiliations:** 1 Library of China Medical University, Shenyang, Liaoning China; 2 Department of Social Medicine, School of Public Health, China Medical University, Shenyang, Liaoning China; 3 First Affiliated Hospital, China Medical University, Shenyang, Liaoning China

**Keywords:** Symptoms of depression, Symptoms of anxiety, Perceived stress, Hope, Resilience, Ovarian cancer patients

## Abstract

**Background:**

It is well known that cancer patients tend to have high levels of perceived stress and symptoms of depression and anxiety. However, there is less study on the association between perceived stress and symptoms of depression and anxiety among ovarian cancer patients in China. And the mediating effect of hope and resilience needs to be further studied. In this study, we aim to examine the prevalence of depression and anxiety symptoms, to analyze the association between perceived stress and symptoms of depression and anxiety, and to test whether hope and resilience mediate the association of perceived stress with symptoms of depression and anxiety among Chinese patients with ovarian cancer.

**Method:**

A total of 220 questionnaires were distributed and collected from the First Affiliated Hospital of China Medical University and Shengjing Hospital of China Medical University. All participants in this study were ovarian cancer inpatients. After excluding the incomplete questionnaires, 198 questionnaires were valid for the analysis. Qualified patients were asked to response to the questionnaires including Hospital anxiety and depression scale (HADS), perceived stress scale (PSS-10), and the Herth hope scale and the resilience scale. Hierarchical regression analyses were used to test the associations among perceived stress, symptoms of depression and anxiety, and hope and resilience. Bootstrapping method was conducted to examine whether the indirect effect of hope and resilience was significant respectively.

**Results:**

The prevalence of symptoms of depression and anxiety in ovarian cancer patients was 47.0% and 51.5% respectively. Perceived stress correlated significantly with symptoms of depression (*r* = 0.709, *P* < 0.01) and anxiety (*r* = 0.660, *P* < 0.01). Hope (a*b = 0.155, BCa 95% CI: 0.094, 0.223) partly mediated the association between perceived stress and symptoms of depression; similarly, hope (a*b = 0.129, BCa 95% CI: 0.048, 0.205) partly mediated the effect of perceived stress on symptoms of anxiety. However, resilience (a*b = 0.004, BCa 95% CI: -0.030, 0.040) did not mediate the association between perceived stress and symptoms of depression. And resilience (a*b = 0.041,BCa 95% CI: -0.013, 0.098) did not mediate the association between perceived stress and symptoms of anxiety.

**Conclusions:**

The present study suggests that perceived stress might be one of the impact factors of symptoms of depression and anxiety, while hope might ease symptoms of depression and anxiety. In view of the role of hope, medical workers and patient caregivers should pay more attention to hope, and then to intervene perceived stress among patients with ovarian cancer.

## Background

The incidence and mortality of ovarian cancer varies in different countries. It was estimated that, in 2016, there would be about 22,280 newly diagnosed cases of ovarian cancer and the death from ovarian cancer would be 14,240 in the United States [1]. It was forecasted that nearly 1480 females would be diagnosed with ovarian cancer in Australia, and approximately 1040 females would die of the disease in 2016. [2]. It was reported that Asian countries had diagnosed 110,526 cases of ovarian cancer, and China was the country with the highest number of cases (34,575 cases) [3]. As has been investigated, ovarian cancer is the first leading cause of death from gynecological cancers in China [4]. However, it is difficult to diagnose ovarian cancer at early stages (I/II) for most symptoms are nonspecific. Most of the patients with ovarian cancer are often diagnosed at advanced stages [5, 6]. Therefore, it is imperative to emphasize on ovarian cancer patients.

It is well known that cancer is a kind of disease which threatens one’s health and lives severely. Patients who are suffering from cancer often have to tolerate not only physical pain, but also enormous emotional pressure and huge financial burden [7–12]. Some studies have indicated that symptoms of depression and anxiety were two common types of psychological disorders to cancer patients [13–16]. For example, the diagnosis and treatment of cancer is now mainly regarded as life stress, and this stress could cause or exacerbate relative psychological disorders. Furthermore, a study of meta-analysis has shown that the incidence (54.90% and 49.69%) of symptoms of depression and anxiety in Chinese cancer patients is obviously higher than that in the patients without cancer (17.50% and 18.37%) [17]. More notably, unrealized or untreated symptoms of depression and anxiety could adversely impact the control of morbidity, the adherence with treatment, the healing of illness, and the well-being of life quality [18–20].Thus, discovery and intervention of psychological symptoms of depression and anxiety and stress among cancer patients is considered as a very important topic to the local health authority.

It indicated that 30% to 70% ovarian cancer patients had varying degrees of anxiety syndromes [21, 22], and around 50% patients had clinical depressive symptoms [23]. The patients had to re-handle their relationship with their family members, friends and boss, and they were compelled to face with unknown disaster in job, family income, and lives. However, there are few studies concerning the level of prevalence of symptoms of depression and anxiety in Chinese patients who have been diagnosed with ovarian cancer.

In recent years, positive psychology interventions have been explored by scientists to prevent mental disorders, and conquer disease, achieving a satisfactory life expectancy among oncology patients. Positive psychological resources include many variables such as self-efficacy, hope, resilience, optimism, self-esteem, and social support. Our previous studies have assessed the association of several positive psychological factors with symptoms of depression and anxiety in cervical cancer patients [24] or in newly diagnosed bladder/kidney cancer patients [25]. The present study aims to further assess the effect of perceived stress and two kinds of positive psychological variables (hope and resilience) on symptoms of depression and anxiety in ovarian patients.

For cancer patients, hope is a faith to conquer the disease. Individuals, who maintain high level of hope, will probably be more active in seeking treatment and will have more chances to recovery. Psychological resilience means effective response to a negative event. Cancer patients must constantly withstand a wide range of pressures to conquer or live with cancer. Thus psychological resilience is a vital ability. Therefore, hope and resilience are two indispensable abilities and psychological characteristics in the process of facing cancer. Several studies have provided evidence for the influence of positive psychological factors in cancer patients [25–28]. A literature suggested that higher level of hope predicted greater patient marital satisfaction among couples with advanced breast cancer [28]. Higher psychological flexibility significantly contributed to predict lower level of symptoms of depression and anxiety in patients with breast cancer [29]. A study indicated that psychological resilience, rather than hope, significantly associated with symptoms of depression and anxiety among early-stage bladder/kidney cancer patients [25]. However, whether hope and resilience associated with perceived stress, symptoms of depression and anxiety in ovarian cancer patients remains to be investigated further.

The aim of the study was to assess (1) the prevalence of symptoms of depression and anxiety in ovarian cancer patients from China. (2) Whether perceived stress is associated with symptoms of depression and anxiety in ovarian cancer patients. (3) The mediating role of hope and resilience between perceived stress and symptoms of depression and anxiety in ovarian cancer patients.

## Methods

### Ethics statement

The Committee for Human Trials of China Medical University has reviewed and provided the ethical approval for this study, and the trial procedures were in accordance with the ethical standards. All the patients have submitted their written consent after learning the study protocol. They were all voluntary and anonymous during the process. We protected the privacy of patients in dealing with personal data and maintained confidentiality of individual records.

### Study design and recruitment criteria

From January 1, 2014 to December 31, 2015, we conducted a convenience sampling. We recruited patients with ovarian cancer from the First Affiliated Hospital of China Medical University and Shengjing Hospital of China Medical University, the two tertiary referral hospitals, which are important providers of cancer treatment services in Liaoning Province of China. The eligibility criteria for patient recruitment were (1) at least 18 years old; (2) being pathologically proven ovarian cancer; (3) aware of the cancer diagnosis; (4) able to communicate and answer the questionnaires in Chinese easily; (5) with clear consciousness and cognition. Exclusion criteria were (1) patients had a history of psychiatric problems (e.g. symptoms of depression or anxiety, or other psychiatric disorders) before cancer diagnosis; (2) patients had intellectual impairments; (3) patients had other active cancers.

The questionnaires were filled in the inpatient departments. All eligible patients were invited to participate by their oncologists or physicians. In principle, it is the ovarian cancer patient herself that fills out the questionnaire including demographic questions, Hospital anxiety and depression scale (HADS), perceived stress scale (PSS-10), the Herth hope scale and the resilience scale. The doctors reported the clinical variables according to the records of patients. If the patient is too old to need help, the doctor will read the questions and write the selection according to the patient’s oral report without making any suggestions. After the patients were well-informed about the study, they began to fill in the questionnaire. Initially, a total of 220 patients were enrolled. Finally, we received effective responses from 198 ovarian cancer patients, and the effective response rate is 90%.

### Measurements of symptoms of depression and anxiety

We choose Hospital Anxiety and Depression Scale (HADS) [30] to measure the degree of symptoms of depression and anxiety. This is a 14-item questionnaire including depression subscales (seven entries) and anxiety subscales (seven entries). Each item is rated on a four-point Likert scale (0 = completely not; 1 = a little bit; 2 = somewhat and 3 = very much). The total score of each subscale ranges from zero to 21 points. The higher the score is, the more significant symptom of anxiety and depression is. Zigmonfd and Snaith have recommended the cut-off values for both symptoms of depression and anxiety [30]. Each person is grouped according to a classification where a score of “less than 8” is within normal range, “8–10” indicates a possible clinical anxiety / depression and “more than 10” suggests a probable anxiety / depression mood disorder. The Chinese version of HADS has been widely used in previous studies with sufficient reliability [31, 32]. The internal reliability alpha valued for the symptoms of depression and anxiety in the current study were 0.848 and 0.764 respectively.

### Measurement of perceived stress

Perceived stress was assessed with the Chinese version of Perceived Stress Scale (PSS-10) [33]. It is a 10-item questionnaire whose each item is rated on a 5-point Likert scale ranging from “never” to “always” (0 = Never so, 1 = hardly so, 2 = sometimes, 3 = so often, 4 = always.). Higher total score indicates higher level of pressure that the individual feels. Chinese version of PSS-10 has been used in Chinese population and demonstrated with sufficient reliability [34]. In our study, the Cronbach’s alpha for the total scale was 0.854.

### Measurement of hope

The Herth Hope Index (HHI) was used to assess patients’ overall level of hope [35]. It is a questionnaire including 12 items. Each item is rated on a 4-point Likert scale (1 = strongly disagree; 4 = strongly agree) and total scores range from 12 to 48. Higher score reflects higher level of hope. In our study, Cronbach’s alpha coefficient for the total scale was 0.840.

### Measurement of resilience

The Resilience Scale (RS), developed by Nian Wagnild, was used to assess resilience [36]. The RS scale comprises 14 items. Each item was answered by using a 7-point Likert-type scale (1 = strongly disagree; 7 = strongly agree). Total score ranges from 14 to 98. Higher scores indicate higher level of resilience. The Chinese Resilience Scale has a good content reliability [37]. In this present study, Cronbach’s alpha coefficient of total scale was 0.903.

### Demographic and clinical characteristics

In our study, there are four demographic variables and four clinical variables. Age was divided into three types: “≤45”, “46–55”, and “≥56”. Marital status was divided into two cases: “Married/living with a partner”, “Single/widowed/divorced”. Education level was divided into four levels: “Primary school”, “Middle school”, “High school”, “Junior college or above”. Family income (RMB: Yuan) included “≤1000”, “1001–2000”, “2001–3000”, “3001–4000”, and “≥4001”. According to the International Federation of Gynecology and Obstetrics (FIGO) [38, 39], the study divided cancer stage into three types. Treatment type included “no treatment”, “chemotherapy”, “surgery”, and “combined treatment (a combination of different kinds of treatment)”. Whether the cancer is metastasis is also considered as a clinical variable.

### Statistical analysis

**Fig. 1:**
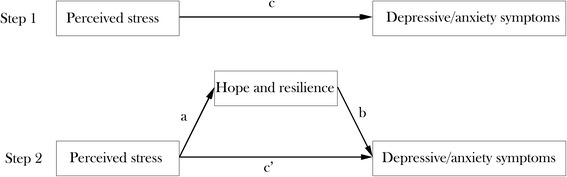
Theoretical model of the mediating role of hope and resilience. (**a**) the relationship between perceived stress and hope and resilience; (**b**) the relationship of hope and resilience with depressive/anxiety symptoms after controlling the independent variables; (**c**’) the association between perceived stress and depressive/anxiety symptoms after adding hope and resilience as mediators

All analysis was conducted by SPSS 17.0 for Windows. We used t-test and one-way ANOVA analysis to compare the difference of symptoms of depression and anxiety according to demographic and clinical groups. In addition, all statistical tests were two-sided (α = 0.05). We performed hierarchical linear regression analysis which tests the mediating effect by three steps. In the first step, the demographic and clinical variables were added. And we set dummy variables for the discontinuous variables. In the second step, perceived stress was added. In the third step, hope and resilience was added. In addition, we used bootstrapping method to examine whether the indirect effect of hope and resilience was significant. Perceived stress was modeled as independent variable, with symptoms of depression and anxiety as the outcomes, hope and resilience as mediators (as shown in Figure 1), and age and treatment type as covariates. The “c path” refers to the relationship between perceived stress and symptoms of depression and anxiety; the “a × b path” represents the mediation of hope and resilience. If the absolute value of “c’ path” coefficiently shrinks than that of the “c path”, the mediation role of hope and resilience may exist. Five thousand bootstrap samples were used to estimate the present study. We suppose that if bias-corrected and accelerated 95% confidence interval (BCa 95% CI) do not include 0, the mediation is significant.

## Results

### Patients’ characteristics

Table 1 presented the patients’ characteristics. The age of the patients (*N* = 198) ranges from 29 to 79 (Mean ± SD: 55.71 ± 9.03). Most of the patients (90.9%) were married or living with a partner. There are 72.8% of patients graduated from middle school or above. And only 18 patients (9.1%) earn more than 4000 Yuan per month. In relation to clinical variables, majority of patients (72.7%) were diagnosed at cancer stage III + IV, and 93.4% received chemotherapy or surgery, or combined treatment. 92.9% were free of metastases.

**Table 1:** Demographic and clinical variables of participants (*N* = 198)

Variable	N	%	Mean ± SD	Median (Range)
Demographic variables	198			
Age (years)			55.71 ± 9.03	55 (29–79)
≤45	19	9.6		
46–55	82	41.4		
≥ 56	97	49.0		
Marital status				
Married/living with a partner	180	90.9		
Single/widowed/divorced	18	9.1		
Educational level				
Primary school	54	27.2		
Middle school	73	36.9		
High school	45	22.7		
Junior college or above	26	13.1		
Income (Yuan per month)				
≤1000	27	13.6		
1001–2000	70	35.3		
2001–3000	59	29.8		
3001–4000	24	12.1		
≥ 4001	18	9.1		
Clinical variables				
Cancer stage				
I	35	17.7		
II	19	9.6		
III + IV	144	72.7		
Treatment type				
No treatment	13	6.6		
Chemotherapy	59	29.8		
Surgery	12	6.0		
Combined treatment	114	57.6		
Metastasis				
No	184	92.9		
Yes	14	7.1		
HADS-Depression symptoms	93	47.0	7.01 ± 3.77	7 (0–21)
8–10	63	31.8		
≥ 11	30	15.2		
HADS-Anxiety symptoms	102	51.5	7.37 ± 4.17	8 (0–20)
8–10	60	30.3		
≥ 11	42	21.2		

*HADS* Hospital Anxiety and Depression Scale, *SD* Standard deviation

Table 2 provided the levels of symptoms of depression and anxiety, perceived stress, and hope and resilience. According to the cut-off values of Zigmond and Snaith [40], patients reported 8–10 HADS score were at the border level which is possible case; patients reported 11–21 HADS score were at high likelihood level which is probable case. The prevalence of symptoms of depression and anxiety in ovarian cancer patients was 47.0% (possible cases: 31.8%; probable cases: 15.2%) and 51.5% (possible cases: 30.3%; probable cases: 21.2%) respectively (*N* = 198). The mean scores of symptoms of depression and anxiety were 7.01 ± 3.77 and 7.37 ± 4.17. The mean values were 17.97 ± 4.83 for perceived stress, 35.44 ± 4.02 for hope and 37.20 ± 7.15 for resilience.

**Table 2:** Descriptive statistics for symptoms of depression and anxiety, perceived stress, hope, and resilience (*N* = 198)

Variables	Mean	SD	Range	N (%)
HADS-Depression symptoms	7.01	3.77	0–21	93 (47.0)
8–10				63 (31.8)
≥ 11				30 (15.2)
HADS-Anxiety symptoms	7.37	4.17	0–20	102 (51.5)
8–10				60 (30.3)
≥ 11				42 (21.2)
PSS	17.97	4.83	4–30	
HHI	35.44	4.02	26–47	
RS	37.20	7.15	22–56	

*HADS* Hospital Anxiety and Depression Scale, *HHI* Herth Hope Index, *PSS* Perceived Stress, *RS* Resilience, *SD* Standard deviation

### Associations of demographic and clinical variables with symptoms of depression and anxiety

As shown in Table 3, results revealed the symptoms of depression and anxiety scores in demographic and clinical variables. Educational level and cancer stage were significantly associated with symptoms of depression; educational level, income and cancer stage were significantly associated with symptoms of anxiety (*p* < 0.05). In addition, patients whose educational level was junior college or above had a lower level of symptoms of depression and anxiety than the patients whose educational level was high school or lower; Or in other words, the higher the educational level, the lower the level of symptoms of depression and anxiety is. Moreover, patients whose cancer stage was ‘I’ had a lower level of symptoms of depression and anxiety than those whose cancer stage was ‘III’ + ‘IV’ or ‘II’. Furthermore, patients whose income per month was 1000 or more lower had higher level of symptoms of anxiety than the others.

**Table 3:** Mean scores of symptoms of depression and anxiety according to demographic and clinical variables

Variables	HADS-Depression symptoms	F/t value	*P* value	HADS-Anxiety symptoms	F/t value	*P* value
Demographic variables						
Age (years)		2.928	0.056		1.77	0.173
≤45	5.53 ± 3.41			5.84 ± 3.50		
46–55	6.68 ± 3.74			7.26 ± 4.20		
≥ 56	7.58 ± 3.79			7.77 ± 4.23		
Marital status		0.075	0.785		0.138	0.711
Married/living with a partner	7.03 ± 3.88			7.34 ± 4.23		
Single/widowed/divorced	6.78 ± 2.62			7.72 ± 3.63		
Educational level		5.049	0.002		3.422	0.018
Primary school	8.26 ± 3.34			8.17 ± 3.95		
Middle school	7.10 ± 3.79			7.64 ± 4.08		
High school	6.56 ± 4.04			7.29 ± 4.61		
Junior college or above	4.96 ± 3.23			5.12 ± 3.42		
Income (Yuan per month)		1.668	0.159		2.940	0.022
≤1000	7.37 ± 3.26			9.07 ± 3.97		
1001–2000	7.36 ± 3.83			7.34 ± 4.03		
2001–3000	7.20 ± 4.29			7.29 ± 4.42		
3001–4000	6.67 ± 2.79			7.67 ± 4.36		
≥ 4001	4.94 ± 3.23			4.83 ± 2.73		
Clinical variables						
Cancer stage		5.73	0.004		4.308	0.015
I	5.11 ± 3.08			5.54 ± 2.95		
II	7.05 ± 2.66			8.11 ± 3.73		
III + IV	7.47 ± 3.92			7.72 ± 4.37		
Treatment type		0.930	0.427		1.12	0.344
No treatment	5.54 ± 3.02			5.85 ± 2.67		
Chemotherapy	7.20 ± 3.98			7.83 ± 4.22		
Surgery	6.25 ± 3.02			6.25 ± 3.57		
Combined treatment	7.16 ± 3.81			7.43 ± 4.32		
Metastasis		0.000	0.992		0.000	0.988
No	7.01 ± 3.87			7.38 ± 4.28		
Yes	7.00 ± 2.15			7.36 ± 2.37		

*HADS* Hospital Anxiety and Depression Scale

### Correlations between study variables

Pearson’s correlation coefficients were calculated among perceived press, hope, resilience and symptoms of depression and anxiety. As shown in Table 4, symptoms of depression was negatively associated with the two positive psychological variables (hope: *r* = −0.668, *P* < 0.01; resilience: *r* = −0.373, *P* < 0.01). A similar pattern was also observed between symptoms of anxiety and the two variables (hope: *r* = −0.587, *P* < 0.01; resilience: *r* = −0.406, *P* < 0.01). Perceived Stress was significantly correlated with symptoms of depression (*r* = 0.709, *P* < 0.01) and symptoms of anxiety (*r* = 0.660, *P* < 0.01).

**Table 4:** Correlation among symptoms of depression and anxiety, hope, resilience and perceived stress

Variables	HADS-Depression symptoms	HADS-Anxiety symptoms	HHI	RS	PSS
HADS-Depression symptoms	1	0.812**	−0.668**	−0.373**	0.709**
HADS-Anxiety symptoms		1	−0.587**	−0.406**	0.660**
HHI			1	0.402**	−0.587**
RS				1	−0.478**
PSS					1

*HADS* Hospital Anxiety and Depression Scale, *HHI* Herth Hope Index, *PSS* Perceived Stress, *RS* Resilience

*P < 0.05, **P < 0.01

### Hierarchical regression analysis

Table 5 indicated the results of hierarchical regression analysis of symptoms of depression after controlling demographic and clinical variables. Perceived stress was significantly associated with symptoms of depression. Perceived stress explained 42.2% of the variance in symptoms of depression among ovarian cancer patients. Hope was negatively associated with symptoms of depression (*β* = −0.347, *P* < 0.01) and hope explained 7.4% of the variance in symptoms of depression. When hope and resilience were added, the regression coefficient for perceived stress diminished (from *β* = 0.710 to *β* = 0.498, *P* < 0.01). The results suggest that hope probably mediate the correlation between perceived stress and symptoms of depression partly.

**Table 5:** Hierarchical linear regression for exploring the correlates of symptoms of depression

	HADS-Depression symptoms		
Variables	Step 1(*β*)	Step 2(*β*)	Step 3(*β*)
Block 1			
Age	0.145	0.171^**^	0.124^*^
“Middle school” vs “Primary school”	−0.148	0.007	−0.014
“High school” vs “Primary school”	−0.206^*^	0.022	0.018
“Junior college or above” vs “Primary school”	−0.221^*^	−0.061	−0.065
Cancer stage “II” vs “I”	0.160	0.129^*^	0.112^*^
Cancer stage “III + IV” vs “I”	0.222^*^	0.193^**^	0.163^**^
Block 2			
PSS		0.710^**^	0.498^**^
Block 3			
HHI			−0.347^**^
RS			−0.032
*F*	2.223^**^	15.399^**^	18.486^**^
Adjusted R^2^	0.085	0.539	0.615
△R^2^	0.155^**^	0.422^**^	0.074^**^

*P < 0.05, **P < 0.01

*HADS* Hospital Anxiety and Depression Scale, *HHI* Herth Hope Index, *PSS* Perceived Stress, *RS* Resilience

Then we examined the mediating effect by bootstrapping method. As shown in Table 6, perceived stress had a significant correlation with symptoms of depression. Perceived stress was negatively correlated with hope and resilience (the “a path”). Hope correlated with symptoms of depression negatively and significantly (the “b path”). However, resilience did not show significant correlation with symptoms of depression. BCa 95% CI for a × b of hope excluding 0 indicated its significant mediation when it was added in the model (the “c’ path”). In contrast, BCa 95% CI for a × b of resilience including 0 indicated its insignificant mediation. We used formula (a × b/ c) to calculate the proportion of mediation. The proportion of hope mediating effect was 29.18% for perceived stress.

**Table 6:** Bootstrapping test of the indirect effect whether hope and resilience act as potential mediators in the correlation between perceived stress and symptoms of depression

Mediators	c	a	b	c’	a × b (BCa 95% CI)
HHI	0. 530^**^	−0.479^**^	−0.319^**^	0.373^**^	0.155 (0.094, 0.223)
RS		−0.718^**^	−0.006		0.004 (−0.030, 0.040)

**P < 0.01

Table 7 showed the results of hierarchical regression analysis of symptoms of anxiety after controlling demographic and clinical variables. Perceived stress was significantly associated with symptoms of anxiety. Perceived stress explained 37.5% of the variance in symptoms of anxiety among ovarian cancer patients. Hope was negatively associated with symptoms of anxiety (β = −0.267, *P* < 0.01) and explained 5.8% of the variance in symptoms of anxiety. However, resilience did not show significant correlation with symptoms of anxiety. When hope and resilience were added, the regression coefficient for perceived stress diminished (from β = 0.670 to β = 0.464, *P* < 0.01). That is to say hope may have a partly mediating effect on the relationship between perceived stress and symptoms of anxiety.

**Table 7:** Hierarchical linear regression for exploring the correlates of symptoms of anxiety

	HADS-Anxiety symptoms		
Variables	Step 1(*β*)	Step 2(*β*)	Step 3(*β*)
Block 1			
Age	0.160*	0.185**	0.142*
“Middle school” vs “Primary school”	−0.042	0.105	0.079
“High school” vs “Primary school”	−0.078	0.137	0.139*
“Junior college or above” vs “Primary school”	−0.146	0.004	−0.018
“1001–2000” vs “1000”	−0.190	−0.154	−0.169*
“2001–3000” vs “1000”	−0.155	−0.211*	−0.233**
“3001–4000” vs “1000”	−0.106	−0.156*	−0.129
“4001” vs “1000”	−0.201*	−0.060	−0.035
Cancer stage “II” vs “I”	0.194*	0.165*	0.148*
Cancer stage “III + IV” vs “I”	0.175*	0.148*	0.135*
Block 2			
PSS		0.670**	0.464**
Block 3			
HHI			−0.267**
RS			−0.115
*F*	2.046*	12.217**	13.592**
Adjusted R^2^	0.074	0.477	0.535
△R^2^	0.144*	0.375**	0.058**

*HADS* Hospital Anxiety and Depression Scale, *HHI* Herth Hope Index, *PSS* Perceived Stress, *RS* Resilience

*P < 0.05, **P < 0.01

Then we examined the mediating effect by bootstrapping method. As shown in Table 8, perceived stress had a significant correlation with symptoms of anxiety. Perceived stress negatively correlated with hope and resilience (the “a path”). Hope correlated with symptoms of anxiety negatively and significantly (the “b path”). BCa 95% CI for a × b of hope excluding 0 indicated its significant mediation when it was added in the model (the “c’ path”). In contrast, BCa 95% CI for a × b of resilience including 0 indicated its insignificant mediation. We used formula (a × b/ c) to calculate the proportion of mediation. The proportion of hope mediating effect was 22.89% for perceived stress.

**Table 8:** Bootstrapping test of the indirect effect whether hope and resilience act as potential mediators in the correlation between perceived stress and symptoms of anxiety

Mediators	c	a	b	c’	a × b (BCa 95% CI)
Hope	0.565^**^	−0.471^**^	−0.266^**^	0.400^**^	0.129 (0.048, 0.205)
resilience		−0.696^**^	−0.057		0.041 (−0.013, 0.098)

**P < 0.01

## Discussion

The prevalence of symptoms of depression is 47.0% and the prevalence of symptoms of anxiety is 51.5% in Chinese patients with ovarian cancer.A meta-analysis indicated that the prevalence of symptoms of depression and anxiety in Chinese cancer patients was 54.90% and 49.69% [17]. Mielcarek P [40] assessed Poland patients with advanced ovarian cancer. They found that the level of symptoms of anxiety was higher than the level of symptoms of depression. And the prevalence of pathological anxiety was the highest (74%) prior to surgery. Watts S [41] quoted an evidence of the prevalence of symptoms of depression (25.34%, 22.99%, and 12.71%) and symptoms of anxiety (19.12%, 26.23%, and 27.09%) in ovarian cancer patients across pretreatment, on-treatment and post-treatment in a systematic review and meta-analysis article. Moreover, Melanie A Price [42] had done a prospective cohort study and found the clinical depressive symptoms was 5.9%, clinical anxiety symptoms was 15% in 798 Australian ovarian cancer patients. There is no doubt that population-based sample is one of the factors that may affect the different results among our study and previous studies. In the developed countries, the improved recognition of mental health disorders from ovarian cancer patients may lead them actively to seek for mental health treatment and other support services. However in China, the level of symptoms of depression and anxiety is relatively low. In contrast, the recognition of psychological disease from ovarian cancer patients in the developing countries is still need to be improved.

Our results have provided direct evidence that perceived stress significantly associated with symptoms of depression and anxiety in ovarian cancer patients. Pranjic N [43] who is from Bosnia and Herzegovina pointed out the high level of distress in cancer patients still needs further attention, powerful intervention and effective treatment. A research [44] suggested that cancer-related fatigue correlated with perceived stress, anxiety and pain severity. It is noteworthy that the results also indicated symptoms of anxiety mediate the association between perceived stress and cancer-related fatigue. A few studies concentrate on symptoms of post-traumatic stress disorder and its association with adult attachment and symptoms of depression in ovarian cancer patients [45, 46]. Then, besides symptoms of depression and anxiety which have been mainly studied in our paper, ovarian cancer patients might also have post-traumatic stress disorder or adult attachment in some extent. Hill EM [47] recruited one hundred ovarian cancer patients and assessed the role of social support seeking on mental health including symptoms of depression and stress. However, the author had not assessed the association among stress and symptoms of depression and anxiety. There are several studies showing a beneficial impact of cognitive-behavioral therapy (CBT) on symptoms of depression and anxiety and insomnia experienced by individuals with cancer [48–50].

Our results showed that hope and resilience were negatively associated with symptoms of depression and anxiety among ovarian cancer patients. And hope played a partly mediating role on the association between perceived stress and symptoms of depression. Similarly, hope partly mediated the effect of perceived stress on symptoms of anxiety. Some of our findings were in accordance with previous studies. Sjoquist KM [51] found that trait hope was negatively correlated with symptoms of depression and anxiety in ovarian cancer patients who have done chemotherapy. Since the level of hope may play a significant role in relieving symptoms of depression and anxiety of ovarian cancer patients, it is necessary to attempt to implement some intervention programs or strategies to help patients maintain and foster hope. For example, nurses can help cancer patients to maintain hope by talking with them, showing special warmth and compassion. And the doctor intervention programs will be also important. The doctors should encourage patients to maintain hope, and build long-term relationship with their ovarian cancer patients by social media tools to answer their questions, send them medical advice, and even tweet them inspiring stories.

As for demographic and clinical variables, this study indicated that patients whose education level was primary school, or patients who were diagnosed at in “III + IV” stage had a more higher level of symptoms of depression than the others; patients whose education level was primary school or patients whose income was 1000 Yuan or lower, or patients who was diagnosed at in “III + IV” stage had a higher level of symptoms of anxiety than the others. Similarly, Hall AE [52] reported that demographic characteristics such as financial burden caused by cancer were significant factors affecting the level of symptoms of depression and anxiety among hematological cancer survivors.

There are some limitations: firstly, we used a convenience sample from only two tertiary referral hospitals. Secondly, it is a cross-sectional study from which we could not draw comparable results across time. Thirdly, we only focused on the symptoms of depression and anxiety; other psychological disorders such as post-traumatic stress disorders, obsession and inferiority have not been investigated. Fourthly, in the present study, heterogeneous issues might affect the associations between variables. In the regression analysis, we have controlled the variables that have significant impact such as cancer stage. However, other variables such as treatment type, social status, times of surgery, times of hospitalization have not been controlled. Despite of limitations, we have drawn important evidence on the effect of perceived stress on symptoms of depression and anxiety in Chinese patients with ovarian cancer. We also have tested whether hope and resilience mediate the effect of perceived stress on symptoms of depression and anxiety by bootstrapping method.

## Conclusions

Firstly, the prevalence of symptoms of depression and anxiety in Chinese patients with ovarian cancer was 47.0% (possible cases: 31.8%; probable cases: 15.2%) and 51.5% (possible cases: 30.3%; probable cases: 21.2%) respectively. Educational level and cancer stage were significantly associated with symptoms of depression and anxiety. In addition, income was significantly associated with symptoms of anxiety. Secondly, the results of hierarchical regression analyses suggested that perceived stress was associated with symptoms of depression and anxiety significantly. Thirdly, bootstrapping test suggested that hope partly mediated the effect of perceived stress on symptoms of depression; and similarly hope had a partly mediating effect on the relationship between perceived stress and symptoms of anxiety. Therefore, we should pay more attention to hope, relieving symptoms of depression and anxiety, and then consider intervening perceived stress among patients with ovarian cancer. Furthermore, the effectiveness and mechanism of intervention should be explored in the next study.

## Abbreviations

HADS: Hospital Anxiety and Depression Scale
HHI: The Herth hope Index
PSS: Perceived Stress Scale
RS: The Resilience Scale
